# Intraoperative Iridectomy in Femto-Laser Assisted Smaller-Incision New Generation Implantable Miniature Telescope

**DOI:** 10.3390/jcm13010076

**Published:** 2023-12-22

**Authors:** Rodolfo Mastropasqua, Matteo Gironi, Rossella D’Aloisio, Valentina Pastore, Giacomo Boscia, Luca Vecchiarino, Fabiana Perna, Katia Clemente, Ilaria Palladinetti, Michela Calandra, Marina Piepoli, Annamaria Porreca, Marta Di Nicola, Francesco Boscia

**Affiliations:** 1 Ophthalmology Clinic, Department of Medicine and Science of Ageing, University “G. d’Annunzio” of Chieti-Pescara, Via dei Vestini 31, 66100 Chieti, Italy; rodolfo.mastropasqua@gmail.com (R.M.); matteo.gironi@hotmail.it (M.G.); l.vecchiarino@unich.it (L.V.); fabiana975@hotmail.com (F.P.); katiaclem90@hotmail.it (K.C.); ilaria.palladinetti@libero.it (I.P.); michela.calandra1@gmail.com (M.C.); 2 Eye Clinic, Department of Medical Science, Neuroscience and Sense Organs, University of Bari, 70121 Bari, Italy; valentinapastore@hotmail.it (V.P.); bosciagiacomo@gmail.com (G.B.); marinapiepoli93@gmail.com (M.P.); francescoboscia@hotmail.com (F.B.); 3 International Agency of Prevention of Blindness, 00185 Rome, Italy; 4 Laboratory of Biostatistics, Department of Medical, Oral and Biotechnological Sciences, University “G. d’Annunzio” of Chieti-Pescara, Via dei Vestini 31, 66100 Chieti, Italy; annamaria.porreca@unich.it (A.P.); marta.dinicola@unich.it (M.D.N.)

**Keywords:** small-incision new-generation implantable miniature telescope, age-related macular degeneration, maculopathy, geographic atrophy, visual prosthesis, visual impairment, implantable ophthalmic micro telescope, SING IMT, iridectomy

## Abstract

Background: In this study, we aimed to report the short-term (6 months) effects on visual functionality and safety of femto-laser assisted smaller-incision new-generation implantable miniature telescope (SING-IMT™) implanting, particularly related to postsurgical intraocular pressure increase, in patients suffering from end-stage age-related macular degeneration (AMD) and cataract. This device, designed for monocular use, aims to minimise the impact of the central scotoma by projecting the images onto a larger area of the photoreceptors surrounding the macula. Methods: In this prospective multicentric observational case series study, 6 eyes of 6 patients who underwent SING-IMT™ implantations were enrolled. At baseline and 6 months follow-up, best corrected distance visual acuity (BCDV) and best corrected near visual acuity (BCNVA), intraocular pressure (IOP), anterior chamber depth, endothelial cells count were assessed. In addition, IOP was also measured at 7, 15, 30, 45 days, and at 3 months follow-up. Finally, the incidence of complications was evaluated. Results: At final follow-up, in the study eyes, mean BCDVA improved by +10.0 letters (6.25; 13.8) letters and mean BCNVA improved by −0.30 logMAR (−0.55; −0.20). At postoperative month 6, we reported a mean IOP decrease of 4.50 mmHg (−5.75; −0.25). Interestingly, 83.3% of patients had an increased IOP value in at least one of the first two postoperative follow-ups (7 days and 15 days). In patients in whom intraoperative mechanical iridotomy was not performed, it was necessary to perform a postoperative YAG laser iridotomy to improve IOP management. Compared to the baseline, ECD loss at 6 months follow-up was 12.6%. Conclusions: The SING IMT™ device was found to be effective in the distance and near vision improvement, without serious postoperative complications. We recommend intraoperative mechanical iridectomy in order to easily manage post-operative IOP and to avoid sudden IOP rise with its possible consequences. These good results can be a hope to partially improve the quality of life of patients suffering from severe end stage macular atrophy.

## 1. Introduction

Age-related macular degeneration (AMD) is still a leading cause of central vision loss in developed countries [1]. As one of the main reasons for legal blindness in Western countries, late AMD has a remarkable impact on public health [2,3], affecting 10–13% of over 65 year-old people and with an estimated prevalence of 13.1% and 0.1–0.3% after the age of 85 in European Caucasian and in Asiatic population respectively [4,5,6].

Ageing has a key role as a risk factor for dry-AMD, thus explaining the geographic distribution of disease prevalence [7].

Nowadays, no medical treatment has proven its efficacy for end-stage AMD, which dramatically impacts daily activities and quality of life [8,9].

Extraocular low vision aids have been used for many years in visual rehabilitation settings to enlarge the retinal image of individuals with low vision, including hand/stand magnifiers, handheld telescopes, contact lenses and other tools. However, they showed several limitations in daily life application [10].

Different intraocular implants have been developed as alternative devices for improving quality of life and visual acuity (VA) in AMD as better described in the Supplementary Materials [11,12,13,14,15,16]. The IMT™ works projecting the images onto a larger area of the photoreceptors surrounding the macula.

Based on the positive results of these devices, Samsara SING IMT™ has recently introduced a new IMT™ model, the Smaller-Incision New Generation Implantable Miniature Telescope (SING-IMT™). SING-IMT™ is an in the capsular bag implantable Galilean telescope after lens extraction, providing 2.7× magnification of central VF. It has an ultra-precision wide-angle glass micro-optics mounted on a silicon carrying device, which should be implanted in the capsular bag after removing the natural lenses through a 6.5–7.5 mm clear cut or limbal incision tunnel to extend through the pupil and acquire vertical positioning. It is provided by a preloaded delivery system and a new foldable haptic design to enhance stability and centration.

Recently a large retrospective case series study was published to describe the safety and efficacy outcomes at 3 months follow-up of the SING IMT™ prosthesis [17].

The purpose of this study was to report the short-term effects on visual functionality and safety, at 6 months follow-up, of femto-laser assisted SING-IMT™ implanting in patients suffering from end-stage AMD and cataract.

## 2. Materials and Methods

This prospective multicentric observational case series study was conducted at the Ophthalmology Clinic, University of Chieti-Pescara, Italy and the Ophthalmology Clinic, University of Bari, Italy. This work was approved by the Institutional Review Board, following the principles of the Declaration of Helsinki. The informed consent form was obtained from all patients.

The cases (n = 6) selection was accomplished in May 2022, and patients underwent surgery implantation between June 2022 and March 2023. Inclusion criteria for the enrollment were irreversible late-stage AMD, resulting from dry-type AMD or wet AMD that has been considered stable for at least 6 months, best corrected distance visual acuity (BCDVA) from 20/80 to 20/800 bilaterally, an expectation five-letter improvement using an external telescope, age of 55 years or older with a visual cataract, anterior chamber depth of ≥2.5 mm and axial length > 21 mm. In addition, good peripheral vision in the eye, not receiving an implant, was required. Exclusion criteria for the study were a history of other retinal or retinal vascular diseases, myopia > 6.0 D or hyperopia > 4.0 D in the planned operative eye, unilateral involvement of macular degeneration, uncontrolled glaucoma or IOP > 22 mmHg, steroid-responsive rise in IOP, endothelial cell density (ECD) ≤ 1600 cells/mm^2^, cornea stromal or endothelial dystrophies, including guttae, poor compliance during the preoperative rehabilitation process.

Patients who met the inclusion criteria received additional pre-operative screening by undergoing simulation of the post-operative expected field of view and scotoma reduction effect on visual acuity tested with an external telescope simulator (ETS). Fellow eye is occluded during the test, and it is requested at least a 5 letter improvement in the selected eye, to meet the criteria for implantation.

### 2.1. Preoperative Evaluation

Patients included in the study underwent a complete ophthalmic examination for both eyes, including BCDVA and best corrected near visual acuity (BCNVA), intraocular pressure, axial length, anterior chamber depth, endothelial cells count, corneal thickness, slit-lamp biomicroscopy, fundoscopy, optical coherence tomography biometry, anterior-segment optical coherence tomography (AS-OCT), corneal topography, spectral domain-optical coherence tomography (SD-OCT) and optical coherence tomography angiography OCTA, along with complete medical history. BCVA was measured by the Early Treatment of Diabetic Retinopathy Study (ETDRS) chart from 2 m. Near vision acuity was evaluated using the ETDRS near chart for 40 cm distance. Goldmann applanation tonometer was used for Intraocular pressure (IOP) measurement. Endothelial cells count was obtained with CEM-530 specular microscope (Nidek Co., Ltd., Gamagori, Japan) IOL Master 700 (Carl Zeiss Meditec AG, Jena, Germany) was used for the biometry, axial length and anterior chamber depth. AS-OCT and corneal topography were realized using MS-39 device (CSO, Phoenix software, v.4.1.1.5, Italy) OCT and OCTA examinations were performed using Spectralis^®^ HRA+ OCT (Heidelberg Engineering, Heidelberg, Germany).

### 2.2. Surgical Technique

Topical mydriatic agents were preoperatively administered in order to obtain iris dilation (>5.70 mm). All patients included in the study underwent femtosecond laser-assisted cataract surgery (FLACS) with LenSx^®^Laser System (Alcon, Fort Worth, TX, USA), for anterior capsulotomy, but assisted corneal incision and nuclear fragmentation was not performed. FLACS allows improved precision during corneal incisions and anterior capsulotomy (>5.5 mm diameter). A conjunctival peritomy was performed at the 12 o’clock position, and a 2.75 mm triplanar corneal incision was subsequently created in the upper quadrant. Standard cataract surgery procedure was completed using Alcon Costellation System (Alcon, Fort Worth, TX, USA). After phacoemulsification, the corneal incision was enlarged to 7.5 mm and a cohesive ophthalmic viscosurgical device (OVD) was injected in the anterior chamber (AC) in order to maintain the AC depth stable. Thereafter, we performed the in-the-bag SING-IMT™ implantation using preloaded injector. The parameters of the device are 4.4 mm long, 3.6 mm in diameter and an overall haptic-to-haptic diameter of 10.8 mm. Intraoperative peripheral surgical iridectomy was realized in 3 patients (50%), and no iridectomy was performed in the 3 remaining patients intraoperatively. 10/0 Nylon interrupted sutures were placed to close the corneal incision. Cefuroxime was injected into the anterior chamber. Antibiotic, steroid and cycloplegic eye drops were prescribed to be administered post-operatively. Antibiotic and steroid eye drops were applied for around 1 month postoperatively.

### 2.3. Postoperative Evaluation

Patients underwent complete ophthalmologic examination, including slit-lamp biomicroscopy, fundoscopy and intraocular pressure, at 7, 15, 30, 45 days, and at 3 months. Additional evaluations were performed when necessary. At 6 months all parameters were recorded, by an extensive ophthalmologic examination, as described in the preoperative evaluation. Corneal sutures were removed at the discretion of the examiner. Eventually significant adverse events were registered.

### 2.4. Statistical Analysis

All analyses were performed with the open-source statistical R environment (version 3.4.3, the R Foundation for Statistical Computing, Vienna, Austria). Because the study was not powered to allow for inferential statistical comparisons within groups, the focus of the analysis was to identify possible trends across them by summary descriptive statistics.

## 3. Results

A total of 6 eyes of 6 patients (1 man and 5 women) with mean age of 80 (75.0; 83.5) years were included in the study. The demographics and clinical characteristics of patients are reported in Table 1.

The preoperative mean duration of AMD in the patients was 4.75 (2.62; 8.00) years, and 3 subjects were injected with intravitreal Anti-VEGF at least once.

### 3.1. Functional Parameter: Best-Corrected Visual Acuity for Distance and Near

**Figure 1:**
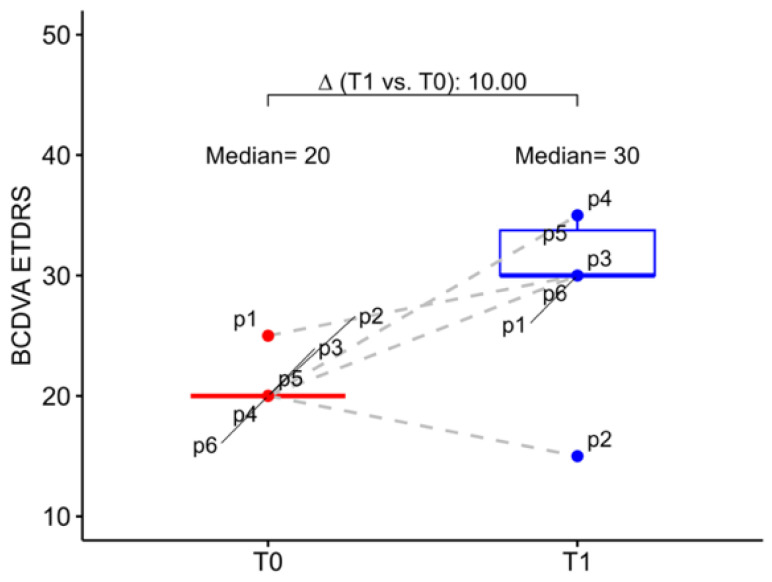
Box–whisker graphs of best corrected distance visual acuity in the patients at evaluation time points (T0) baseline and at 6 months (T1). Box–whisker plots show 25th and 75th percentile range (box) with 95% confidence interval (whiskers) and median values (transverse lines in box). ∆ = absolute differences.

At baseline, the study eye mean BCDVA was 20.00 letters (20.00; 20.00), which corresponds to 1.00 logMAR (1.00; 1.00), while the fellow eyes had a mean BCDVA of 1.30 logMAR (1.09; 1.30). At six months postoperative follow-up mean BCDVA increased, in median, to 30.00 letters (30.00; 33.80) with an improvement of 10.00 letters (6.25; 13.80). In detail, we observed that 5 patients gained at least 1 line on ETDRS chart, 4 patients gained 2 lines or more, 2 patients gained 3 lines or more and 1 patient gained 6 lines, while only one patient had a BCDVA reduction (5 letters) at 6 months follow-up. However, this patient had a worsening of senile dementia in the post-operative period (Figure 1).

**Figure 2:**
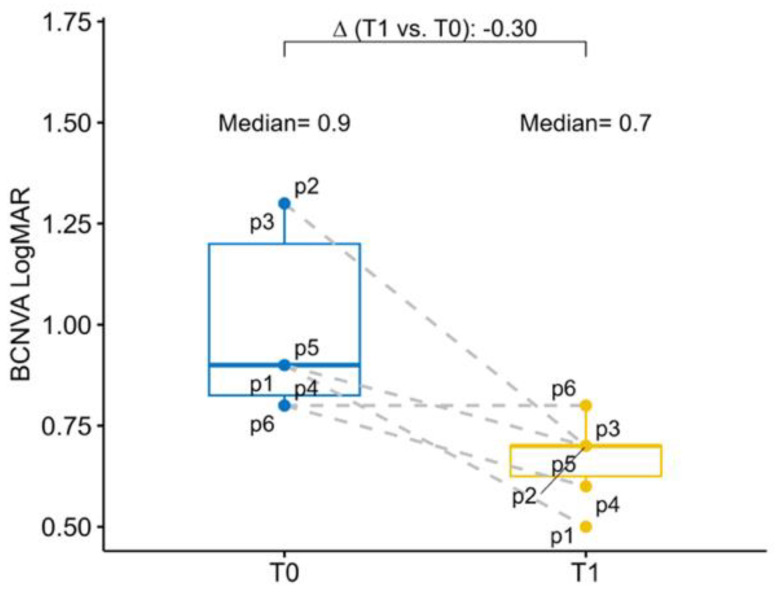
Box–whisker graphs of best corrected near visual acuity in the patients at evaluation time points (T0) baseline and at 6 months (T1). Box–whisker plots show 25th and 75th percentile range (box) with 95% confidence interval (whiskers) and median values (transverse lines in box). ∆ = absolute differences.

The baseline mean BCNVA in the study eye was 0.90 logMAR (0.83; 1.20), while the mean BCNVA in the fellow eye was 1.05 logMAR (1.00; 1.25). At six months postoperative follow-up BCNVA had a mean improvement of −0.30 logMAR (−0.55; −0.20) in the study eye. Overall, all patients have experienced an improvement of the BCNVA (Figure 2).

### 3.2. Anatomical Parameter and Endothelial Cell Density

**Figure 3:**
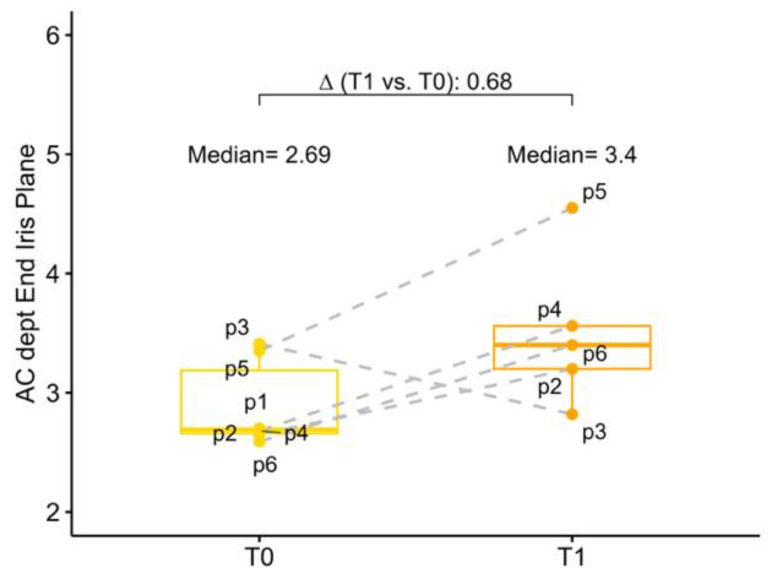
Box–whisker graphs of AC depth at evaluation time points (T0) baseline which correspond to the corneal endothelium-anterior capsule of the crystalline lens distance and at 6 months (T1) in terms of corneal endothelium-interpupillary iris plane distance in the patients. Box–whisker plots show 25th and 75th percentile range (box) with 95% confidence interval (whiskers) and median values (transverse lines in box). ∆ = absolute differences.

**Figure 4:**
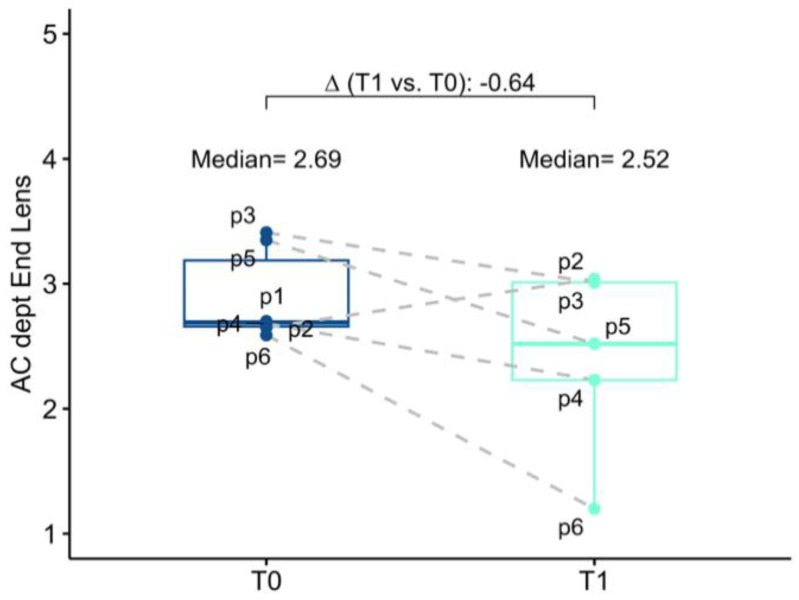
Box–whisker graphs of AC depth at evaluation time points (T0) baseline which correspond to the corneal endothelium-anterior capsule of the crystalline lens distance and at 6 months (T1) in terms of corneal endothelium−telescope distance in the patients. Box–whisker plots show 25th and 75th percentile range (box) with 95% confidence interval (whiskers) and median values (transverse lines in box). ∆ = absolute differences.

The baseline mean AC depth was 2.69 mm (2.66; 3.19) and 3.19 (3.08; 3.28) in the study eye and in the fellow eye, respectively. Anatomical changes after surgery were investigated both in terms of corneal endothelium-telescope distance (EC-IMTd) and in terms of corneal endothelium-interpupillary iris plane distance (EC-IPd). At 6 months follow-up mean EC-IMTd was 2.52 mm (2.23; 3.01), corresponding to a reduction of 0.64 mm (−1.25; −0.41) of AC depth. Six months after surgery mean EC-IPd was 3.40 (3.20; 3.56), representing an increase in depth of 0.68 mm (−0.31; 0.86). In five operated eyes EC-IPd > EC-IMTd, for only one eye EC-IMTd was slightly greater than EC-IPd, meaning that in this case, the telescopic optic was behind the iris plane (Figure 3 and Figure 4).

No differences in post-operative functional or safety outcomes were observed in the patient with the telescopic optic behind the iris plane in comparison to the other study eyes. At the follow-up, fellow eye AC depth was unchanged from baseline.

**Figure 5:**
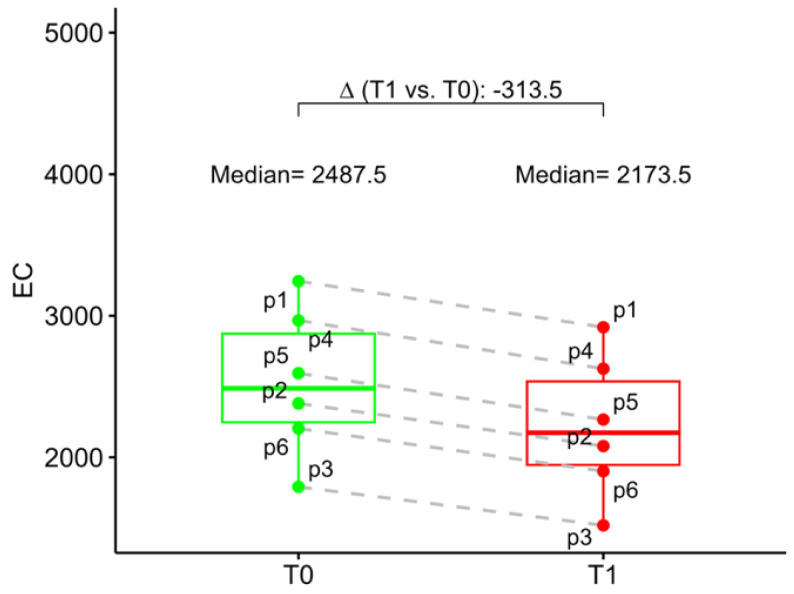
Box–whisker graphs of endothelial cell density in the patients at evaluation time points (T0) baseline and at 6 months (T1). Box–whisker plots show 25th and 75th percentile range (box) with 95% confidence interval (whiskers) and median values (transverse lines in box). ∆ = absolute differences.

At baseline, the mean ECD was 2488 (2248; 2874) and 2542 (2266; 2912) in the study eye and in the fellow eye, respectively. At 6 months follow-up mean study eye ECD was 2174 (1946; 2536). Compared to the baseline, ECD loss at 6 months follow-up was 12.6% (Figure 5).

### 3.3. Intraocular Pressure

The baseline mean IOP was in the study eye 17.5 mmHg (16.2; 18.0). At 6 months follow-up mean IOP was 12.5 mmHg (12.0; 14.5), which represents a decrease of 4.50 mmHg (−5.75; −0.25).

**Figure 6:**
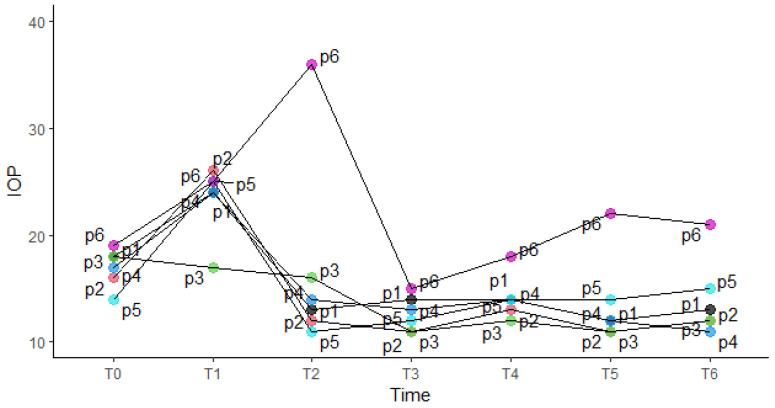
IOP trends at different times for each study eye. T0 = baseline; T1 = 7 days postoperative; T2 = 15 days postoperative; T3 = 30 days postoperative; T4 = 45 days postoperative; T5 = 3 months postoperative; T6 = 6 months postoperative.

As shown in Figure 6, we found an increased IOP in 5 patients at 7-day follow-up, which tends to decrease in four of them during subsequent follow-ups. One patient (P5) underwent YAG laser iridotomy treatment 8 days after surgery due to a sudden increase in blood pressure. One patient (P6) showed a trend of increasing IOP at the 15-day follow-up and underwent YAG laser iridotomy treatment the same day. Thirty and 45 days postoperatively all patients reported a IOP lower than or equal to the preoperative one. At 3- and 6-month follow-ups, only one patient (P6) has higher IOP than in preoperative time (>21 mmHg) (Figure 6).

Another patient (P4) underwent YAG laser iridotomy treatment 59 days after surgery due to an abrupt spike in IOP. Finally, all 3 patients (P4, P5, P6) who did not undergo intraoperative mechanical iridectomy required postoperative YAG laser iridotomy treatment for IOP management. Furthermore, 2 patients (P3 and P4) required post-operative prolonged topical glaucoma therapy, one of which (P4) for 3 months, while the other (P6) at the 6-month follow-up was still on therapy.

### 3.4. Safety Outcomes

No intraoperative complications have been reported. In post-operative time, common reported adverse events were corneal edema, which occurred in 2 patients, but persisted beyond 1 month after surgery in only one (P6), despite topical steroid therapy. One patient (P3) reported postoperative diplopia. As already mentioned, 3 patients required YAG laser iridotomy treatment for postoperative IOP management and 2 of them underwent prolonged topical glaucoma therapy. Finally, one patient (P6) was scheduled for surgical removal of the IMT™, due to poor IOP control consequent to the formation of irido-device synechiae for 360°.

## 4. Discussion

In this prospective multicentric case series, we evaluated functional and anatomical outcomes after SING IMT™ implantation in 6 patients. To the best of our knowledge, it is the first mid-term case series showing data after 6-month follow-up of SING IMT™ device implantation using FLACS surgery.

The largest analysis on the results of Galilean implantable miniature telescope for the treatment of dry AMD was conducted by the pilot study IMT-002, with an end-point at 24 months, and its subsequent extension to 60 months, study IMT-002-LT [12,14]. The authors reported a mean BCDVA improvement of 3.2 lines at 24 months and 2.4 lines at 60 months with first-generation IMT. Similarly, in our experience we found an average increase of 10.0 ETDRS letters (6.25; 13.8) at 6 months after surgery, corresponding to an improvement of 2 lines, but using new-generation IMT (SING-IMT™). On the other hand, if we do not consider the patient who showed post-operative dementia, who is the only one with vision worsening after surgery, the average BCDVA increase in our study population was 14 letters, which corresponded approximately to 3 lines. The age-stratified 12-month results of IMT-002 study highlighted as 79.8% of 75 years-old or elderly patients gained ≥2 lines. Considering the mean age of our study population, which was 80.0 (75.0; 83.5] years old, our findings revealed a BCDVA gain of at least 2 ETDRS lines in 66.7% of patients, which is 80% of patients, if we exclude from the analysis the one affected by dementia. A short-term retrospective case series on SING-IMT™ implants in 24 eyes was recently performed [17]. At three-months end-point follow-up, it reported a BCDVA improvements of ≥ 2 lines in the 70.83% of patients, which is comparable with our results. Despite this, it reported ≥ 3 lines gain in 58.33% of the implanted eye, while in our experience, only 2 patients out of 6 improved ≥ 3 lines on BCDVA. A possible explanation could be the elderly age range of our population (74–86), in fact, the 2 patients who gained 3 lines were respectively 74 and 78 years old, while only 1 of the 3 subjects aged over 80 increased the BCDVA by at least 10 letters.

Of note, duration of AMD seems to be a determining factor for the BCDVA improvement because none of the patients affected by the maculopathy for more than 5 years gained 3 letters.

All the patients of our study reported an improvement in BCNVA in the study eye at 6 months follow-up. Mean BCNVA improvements is −0.30 logMAR, corresponding to 3 lines ETDRS improvement for near vision. This visual improvement was comparable to that described in IMT-002 study showing 3.18 lines gained at 12 months and to that showed in Savastano’s workreporting 3 month-results [12,13].

We aimed at investigating safety outcomes, as well. In literature the most common reported AE were inflammatory deposits on the device [12,13,14,15,18]. Differently, we did not find any inflammatory or pigmented deposits on the lens in our study population. Another frequent complication described in previous studies was corneal edema. IMT-002 study revealed a cumulative incidence of corneal edema of 7% within 30 days after implantation [14]. The incidence of corneal edema in our study was 33.3% (2/6), comparable with 29.2% rate of postoperative corneal edema reported by Toro et al. in their work on SING-IMT™ [18]. Elderly age range of our sample is a possible explanation for the higher rate of edema, also considering that in previous studies on first-generation IMT the rate of corneal edema increased with age of patients (7.1% in >75YO versus 4.3% in <75YO) within 30 days. The most common ocular complication highlighted from our findings was the post-operative IOP raise. First generation IMT™ safety evaluation revealed a cumulative incidence of treatment-needed IOP increasing of 28% within 7 days, while studies on SING-IMT™ reported a rate of 4.2% [14,18]. In our study cohort, 3 patients required post-operative IOP treatments, and two of them for at least 3 months consecutively. All the 3 eyes who experienced treatment needed IOP increasing had not undergone mechanical iridectomy during the surgery. Furthermore, we observed post-operative sudden rise of IOP, at different times, in all the 3 patients without intra-operative iridectomy, who required YAG laser iridotomy. In literature, post-operative iridotomy treatment was not reported to be needed to manage the abrupt rise of IOP, apart from one case in Savastano’s work [13]. Comparing the IOP trends of patients underwent intraoperative iridectomy with others, we strongly suggest performing mechanical iridectomy during the surgery.

The mean loss of ECD rate with IMT™ was previously reported as 20% at 3 months, 13% to 25% at 1 year, and 29% at 2 years [14,15]. Previous study on SING-IMT™ reported a mean ECD decrease of 10.4% at 3 months [18]. We found a mean ECD loss of 12.6% at 6 months in the study eyes, similar to Toro et al. [18] findings. The main advantage of SING IMT™, if compared with the first generations IMT™, is the new device design and technique, allowing small corneal wound, the preloaded device, as well as the shorter surgery time. Further advantages expected from the use of FLACS could not be evaluated because we did not use assisted nuclear fragmentation and it is clearly known that the reduction of postoperative ECD loss is mainly due to a reduced cumulative dissipated energy during laser-assisted phacoemulsification [19]. However, the possibility of setting pre-operatively some anatomical parameters (e.g., the diameter of the capsulorhexis) increases surgical repeatability, predictability, shape accuration and strength of capsulorhexis, as well as reducing the risk of complications, compared to the manual procedure [20]. Moreover, FLACS was related to a lower overall variability of anterior chamber depth compared to conventional cataract surgery, with less postoperative IOL axial movements compared to the conventional tecnique [20,21].

The rate of IMT™ explantation is 5.8% (12/206) on 24-month follow-up [15]. In our study, only one patient was referred for SING-IMT™ explantation after 6-month follow-up. Age stratification identified elderly age as a possible predisposing factor to explantation, indeed 10 out of 12 patients who underwent this procedure were in the over 75 year old group [15]. Despite this, only 2 patients underwent explantation because of dissatisfaction.

## 5. Conclusions

The SING IMT™ device was effective in improving distance and near vision, without serious postoperative complications. These good results can be a hope to partially improve the quality of life of patients suffering from severe end stage macular atrophy. Despite this, a careful preoperative selection of patients is essential for maximizing the potential of the device, particularly considering the age and the duration of the disease. Further studies are needed to validate the long-term safety profile of the SING IMT™. We recommend intraoperative mechanical iridectomy in order to easily manage post-operative IOP and to avoid sudden IOP rise with its possible consequences. FLACS assistance during the surgery seems to be non-influential on the final outcomes. The limited size of the study cohort and the duration of the follow-up are the main limitations of this case series. However, it is a good starting point to better observe long-term functional results in such chronic and irreversible dry end-stage AMD conditions. Undoubtedly, a wider sample and a longer follow up could further strengthen our preliminary data.

## Figures and Tables

**Table 1:** Baseline demographics and clinical characteristics of cases.

Variables	
Patients, n	6
Age, median years (q1; q3)	80.0 (75.0; 83.5)
Male/Female, n	1/5
Right eye/Left eye, n	2/4
Duration of AMD, median yrs (q1; q3)	4.75 (2.62; 8.00)

## Data Availability

All data will be available on request to the corresponding author.

## Supplementary Materials

The following supporting information can be downloaded at: https://www.mdpi.com/article/10.3390/jcm13010076/s1.
