# Supplementary material for: Intraoperative Iridectomy in Femto-Laser Assisted Smaller-Incision New Generation Implantable Miniature Telescope
Source: J Clin Med. 2023 Dec 22;13(1):76. doi: 10.3390/jcm13010076 (PMC10779865; doi:10.3390/jcm13010076)
Supplement: Supplementary file 1 [file jcm-13-00076-s001.zip › jcm-2731107-supplementary.pdf]

Supplementary Materials

The first intraocular Implantable Miniature Telescope (IMT™), presented by Dr Isaac Lipshitz, was designed to provide high magnification for patients affected by macular diseases [11]. Over time, visual functionality positive results lead to the development of new models of IMT™ and other galenic implantable systems [12–14].

The IMT™ works projecting the images onto a larger area of the photoreceptors surrounding the macula. This device, designed for monocular use, aims to minimize the impact of the central scotoma. It consists of an ultra-precise wide-angle micro-optics which, combined with the cornea, work like a fixed-focus telephoto lens. The central Visual Field (VF) of an implanted eye is magnified 2.2–3× (depending on the model) with a 20–24° field of view projected onto approximately 55° of the retina. Not treated eye is usually used for peripheral vision [15].

Post-operative training and support with extended visual rehabilitation (almost 6–12 months) are essential for the patient's adequate use of the device, also because the patient's brain needs time to properly learn efficiently how to use each eye differently for a specific task [16].

1. Lipshitz, I.; Sheah, A.; Loewnstein, A. The Implantable Miniaturized Telescope for patients with age-related macular degeneration: Design and surgical technique. *Oper. Tech. Cataract. Refract. Surg.* **2000**, *3*, 53–58.
2. Hudson, H.L.; Lane, S.S.; Heier, J.S.; Stulting, R.D.; Singerman, L.; Lichter, P.R.; Sternberg, P.; Chang, D.F.; IMT-002 Study Group. Implantable miniature telescope for the treatment of visual acuity loss resulting from end-stage age-related macular degeneration: 1-year results. *Ophthalmology* **2006**, *113*, 1987–2001.
3. Savastano, A.; Ferrara, S.; Sasso, P.; Savastano, M.C.; Crincoli, E.; Caporossi, T.; De Vico, U.; Vidal Aroca, F.; Francione, G.; Sammarco, L.; et al. Smaller-Incision new-generation implantable miniature telescope: Three-months follow-up study. *Eur. J. Ophthalmol.* **2023**, 11206721231212545.
4. Hudson, H.L.; Stulting, R.D.; Heier, J.S.; Lane, S.S.; Chang, D.F.; Singerman, L.J.; Bradford, C.A.; Leonard, R.E.; IMT002 Study Group. Implantable telescope for end-stage age-related macular degeneration: Long-term visual acuity and safety outcomes. *Am. J. Ophthalmol.* **2008**, *146*, 664–673.e1.
5. Boyer, D.; Freund, K.B.; Regillo, C.; Levy, M.H.; Garg, S. Longterm (60-month) results for the implantable miniature telescope: Efficacy and safety outcomes stratified by age in patients with end-stage age-related macular degeneration. *Clin. Ophthalmol.* **2015**, *9*, 1099–107.
6. Grzybowski, A.; Wang, J.; Mao, F.; Wang, D.; Wang, N. Intraocular vision-improving devices in age-related macular degeneration. *Ann. Transl. Med.* **2020**, *8*, 1549.
